# Mortgage Borrowing and Chronic Disease Outcomes in Older Age: Evidence From Biomarker Data in the HRS

**DOI:** 10.1093/geroni/igaf122.1413

**Published:** 2025-12-31

**Authors:** Cäzilia Loibl, Alec Rhodes, Stephanie Moulton, Donald Haurin, Matthew Pesavento, Madison Hyer, Joshua Joseph

**Affiliations:** The Ohio State University, Columbus, Ohio, United States; University of Wisconsin–Madison, Madison, Wisconsin, United States; The Ohio State University, Columbus, Ohio, United States; The Ohio State University, Columbus, Ohio, United States; The Ohio State University, Columbus, Ohio, United States; The Ohio State University, Columbus, Ohio, United States; The Ohio State University, Columbus, Ohio, United States

## Abstract

The medical diagnosis of a disease is common in older age and can carry significant financial costs. For many older adults, equity in a home is their primary component of wealth; however, housing wealth is illiquid. We analyze the relationship between the liquidation of housing wealth through mortgage borrowing on older homeowners’ ability to successfully control a disease. We use data on homeowners aged 65 and older from the 1998–2016 waves of the Health and Retirement Study (N = 3,457). We use biomarkers and physical health indicators to measure disease control following a medical diagnosis of diabetes, heart condition, high blood pressure, lung disease, or cancer. Descriptively, 28% of older homeowners who borrow against home equity are not controlled on their disease, compared to 33% of non-borrowers. Panel data instrumental variable regressions show that each $10,000 borrowed from home equity after diagnosis is associated with a 17-percentage-point reduction in the probability of the disease not being controlled. Many older adults are not able or willing to liquidate housing wealth, and the ability to borrow also depends on changes in home values. Thus, housing wealth is not a uniform social determinant of health but is shaped by older adults’ participation in financial markets. In follow-up research, we have focused on older adults with diabetes and document the role of access to financial resources for the ability of older adults to manage diabetes.

